# Effects of lymphocyte, C-reactive protein and prealbumin levels on clinical typing and course of disease in children infected with novel coronavirus

**DOI:** 10.12669/pjms.38.5.5286

**Published:** 2022

**Authors:** Jing Bi, Yuanda Zhang, Jingzhi Zhang, Qian Han, Chaoyu Ji, Weina Zhen

**Affiliations:** 1 Jing Bi, Department of Infectious Diseases, Baoding Children’s Hospital, Baoding, Hebei, 071000, P.R. China. Baoding Accurate Diagnosis and Treatment Laboratory of Children’s Infectious Diseases, Hebei, 071000, P.R. China; 2 Yuanda Zhang, Department of Gastroenterology, Baoding Children’s Hospital, Baoding, Hebei, 071000, P.R. China; Key Laborary of Clinical Research on Respiratory Digestive Disease, Hebei Baoding, 071000, China; 3 Jingzhi Zhang, Department of Endocrinology, Baoding Children’s Hospital, Baoding, Hebei, 071000, P.R. China; 4 Qian Han, Department of Infectious Diseases, Baoding Children’s Hospital, Baoding, Hebei, 071000, P.R. China. Baoding Accurate Diagnosis and Treatment Laboratory of Children’s Infectious Diseases, Hebei, 071000, P.R. China; 5 Chaoyu Ji, Department of Respiratory Medicine, Baoding Children’s Hospital, Baoding, Hebei, 071000, P.R. China; 6 Weina Zhen, Department of Infectious Diseases, Baoding Children’s Hospital, Baoding, Hebei, 071000, P.R. China. Baoding Accurate Diagnosis and Treatment Laboratory of Children’s Infectious Diseases, Hebei, 071000, P.R. China

**Keywords:** Children, 2019-nCoV, COVID-19, Lymphocyte, Prealbumin, C-reactive Protein

## Abstract

**Objectives:**

To investigate the effects of lymphocyte (LY), C-reactive protein (CRP) and prealbumin (PA) levels on the clinical typing and course of disease in children infected with novel coronavirus (2019-nCoV) at the early stage.

**Methods:**

A total of 140 children with 2019-nCoV infection diagnosed in Shijiazhuang People’s Hospital and Hebei Provincial Chest Hospital from January 2021 to February 2021 were selected for this study. According to the clinical symptoms, laboratory results and imaging examination, the children were divided into asymptomatic infection group, mild infection group and common infection group. The levels of white blood cell (WBC), LY, CRP, PA, albumin (ALB), aspartate aminotransferase (AST), alanine aminotransferase (ALT), creatine kinase (CK) and creatine kinase MB isoenzyme (CKMB) in the children were recorded on the 2nd d after the positive detection of 2019-nCoV nucleic acid.

**Results:**

There were 73(52.1%) children in the asymptomatic infection group, 35(25.0%) children in the mild infection group and 32(22.9%) children in the common infection group. LY level in the common infection group was lower than that in the asymptomatic infection group and the mild infection group (F= 3.152, both *p*< 0.05). CRP level in the common infection group was higher than that in the asymptomatic infection group and the mild infection group (F= 6.343, both *p<* 0.05). CRP level in the mild infection group was higher than that in the asymptomatic infection group (t= 2.052, *p<* 0.05). PA level in the common infection group and the mild infection group was lower compared with the asymptomatic infection group (F= 5.229, both *p<* 0.05). WBC, ALB, AST, ALT, CK and CKMB levels in the three groups showed no statistical significance (F= 1.803, F= 1.208, F= 2.391, F= 1.973, F= 0.401, F= 1.332, respectively, all *p>* 0.05). Correlation analysis demonstrated that LY and PA levels were negatively correlated with hospital stay (r= -0.265, r= -0.325, both *p<* 0.050), but CRP level was not correlated with hospital stay (r= -0.039, *p>* 0.05).

**Conclusion:**

CRP is correlated with the clinical typing of children with 2019-nCoV infection, while LY and PA levels may be closely correlated with the clinical typing and course of treatment of children with 2019-nCoV infection.

## INTRODUCTION

Novel coronavirus pneumonia (COVID-19) caused by novel coronavirus (2019-nCoV) infection is a new acute respiratory infectious disease, which was declared by the World Health Organization (WHO) as a global pandemic in March 2020.^1^-^3^ COVID-19 is mainly transmitted through the respiratory tract. Most of the cases are mild, but some patients suffer from severe pneumonia, endangering their lives. Current studies suggest that patients infected with 2019-nCoV will present normal white blood cell (WBC) count or mild WBC reduction, significant lymphocyte (LY) reduction, increased C-reactive protein (CRP) and decreased prealbumin (PA).^4^-^6^ Moreover, LY, CRP and PA levels are closely related to the severity of COVID-19, which is helpful to evaluate the prognosis of this disease.^7^-^9^

Our objective was to explore whether LY, CRP and PA levels in children with 2019-nCoV infection will change significantly, and whether it will affect the clinical typing and course of disease, the blood routine, hepatic function and myocardial enzymes of 140 children with 2019-nCoV infection were retrospectively analyzed in this study.

## METHODS

A total of 140 children with 2019-nCoV infection diagnosed in Shijiazhuang People’s Hospital and Hebei Provincial Chest Hospital from January 2021 to February 2021 were selected as subjects, including 66 males and 74 females, with an average age of 8.3 ± 3.7 years. The diagnostic and discharge criteria of 2019-nCoV infection referred to the *Expert Consensus on Diagnosis, Treatment and Prevention of 2019-nCoV Infection in Children*.^10^ This study was approved by the ethics committee of Baoding Children’s Hospital.

### Ethical Approval:

The study was approved by the Institutional Ethics Committee of Baoding Children’s Hospital on June 17, 2021 (No.:H-BDETKJ-SOP006-03-A/0), and written informed consent was obtained from all participants

### Clinical typing:

According to the *Expert Consensus on Diagnosis, Treatment and Prevention of 2019-nCoV Infection in Children*,^10^ the children were divided into asymptomatic infection, mild infection, common infection, severe infection and critical infection.

### Observation Indexes:

The general clinical data of the included children were recorded. Additionally, the levels of WBC, LY, CRP, PA, albumin (ALB), aspartate aminotransferase (AST), alanine aminotransferase (ALT), creatine kinase (CK) and creatine kinase MB isoenzyme (CKMB) in the included children were recorded on the 2nd day after the positive detection of 2019-nCoV nucleic acid.

### Specimen collection and detection:

All specimens were collected and detected by qualified personnel. Specimen collection: In the morning, 2-ml fasting venous blood was collected into one purple cap tube and one red cap tube, respectively. The blood in the red cap tube was kept at 4°C-20°C for 30 minutes and centrifuged at 3000 r/min for 10 min, and then the serum was collected for detection. The blood specimens were measured using a XN-2800 full-automatic hematology analyzer (Hysenmek, Japan).The detection was performed using a C800 biochemical analyzer (Roche), and the reagents were purchased from Roche.

### Statistical Analysis:

In this study, the data were analyzed using SPSS 25.0. The measurement data were expressed as (X̄±S), and inter-group comparison was conducted with the analysis of variance and t test, with *p<* 0.05 considered as statistically significant. Correlation analysis was performed using Pearson’s linear correlation analysis, with *p<* 0.05 considered as statistically significant.

## RESULTS

Of the 140 children enrolled in this study, there were 73 (52.1%) children in the asymptomatic infection group, including 35 males and 38 females aged 8.5 ± 3.4 years, 35 (25.0%) children in the mild infection group, including 17 males and 18 females aged 7.2 ± 4.3 years, and 32 (22.9%) children in the common infection group, including 14 males and 18 females aged 9.2 ± 3.6 years. No statistical significance was found in gender or age among the three groups (*p>* 0.05). There were no severe or critical children in the study. LY count reduced in 6 (4.3%) children, CRP increased in 13 (9.3%) children, and PA level decreased in 35 (25%) children.

LY level in the common infection group was lower than that in the asymptomatic infection group and the mild infection group (F= 3.152, *p<* 0.05). LY level showed no statistical significance between the mild infection group and the asymptomatic infection group (t= 0.815, *p>* 0.05). Compared with the asymptomatic infection group and the mild infection group, CRP level in the common infection group was higher (F= 6.343, *p<* 0.05). CRP level in the mild infection group was higher than that in the asymptomatic infection group (t= 2.052, *p<* 0.05). PA level in both common infection group and mild infection group was lower than that in the asymptomatic infection group (F= 5.229, both *p<* 0.05). No statistical significance was found in PA level between the common infection group and the mild infection group (t= 1.329, *p>* 0.05). WBC, ALB, AST, ALT, CK and CKMB levels showed no statistical significance among the three groups (F= 1.803, F= 1.208, F= 2.391, F= 1.973, F= 0.401, F= 1.332, respectively, all *p>* 0.05). Table-I.

**Table I:** Comparison in WBC, LY, CRP, PA, ALB, AST, ALT, CK and CKMB among three groups.

	N	WBC	LY	CRP	PA	ALB	AST	ALT	CK	CKMB
Common infection group	32	6.47±1.89	2.46±1.07	6.17±10.07	154±59	42.6±5.3	33±6	19±10	91±35	28±26
Mild infection group	35	7.28±2.57	3.01±1.81	4.54±8.82	173±62	42.9±2.5	35±11	15±5	99±34	23±7
Asymptomatic infection group	73	6.56±1.73	3.30±1.65	1.42±2.38	185±28	43.6±2.3	31±6	17±10	94±39	23±7
F		1.803	3.152	6.343	5.229	1.208	2.391	1.973	0.401	1.332
P		P=0.169	P=0.046	P=0.002	P=0.006	P=0.302	P=0.095	P=0.143	P=0.670	P=0.267

### Correlation Analysis:

**Fig.1:**
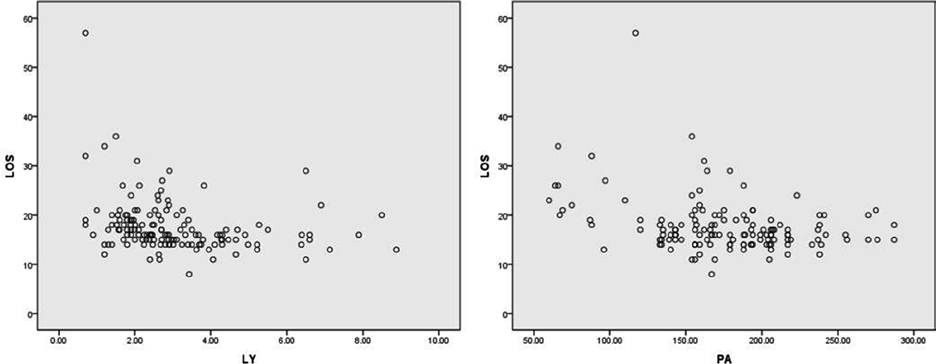
Correlations of LY and PA with hospital stay.

LY and PA levels were negatively correlated with hospital stay (r= -0.265, r= -0.325, respectively, both *p<* 0.050), as shown in Fig.1. CRP level had no correlation with hospital stay (r= -0.039, *p>* 0.05).

## DISCUSSION

By the end of 2019, 2019-nCoV infection-caused COVID-19 was spreading rapidly all over the world. At present, the reported children account for 1%-5% of all confirmed cases of COVID-19.^1^ Compared with adult cases, children with 2019-nCoV infection generally have less clinical symptoms and mild condition, and about 90% infected children are diagnosed as asymptomatic, mild or common type.^11^-^14^ This is consistent with that all the children were asymptomatic, mild or common type, without severe or critical cases, in this study.

Currently, it is believed that blood test indexes such as blood routine have high clinical value for patients with COVID-19.^15^ The increase in CRP and the decrease in LY are closely related to the severity of COVID-19.^6^,^16^-^18^ The results of this study showed that LY was lower while CRP level was higher in the common infection group than those in the asymptomatic infection group and the mild infection group, which is in line with the previous results. People’s Hospital of Wuhan University reported that COVID-19 patients with decreased LY had longer hospital stay.^17^ Our results demonstrated that LY level was negatively correlated with the length of hospital stay in the infected children, suggesting that LY level may be closely related to the clinical typing and course of treatment in children with 2019-nCoV infection. Additionally, this study also analyzed whether there was a correlation between CRP and the length of hospital stay, revealing no correlation, which indicates that the early increase in CRP level may be correlated with the clinical typing in children with 2019-nCoV infection, but not the course of treatment.

In the early stage of infection, patients with COVID-19 may present changes in PA level.^19^ The changes in PA level can be used as an index to determine the severity and progression of COVID-19.^20^ The results of current study showed that the PA level of the common infection group and the mild infection group was both lower than that of the asymptomatic infection group, which is consistent with previous reports. A study has suggested that the level of PA is related to the length of hospital stay.^16^ This study analyzed the correlation between PA level and the length of hospital stay in infected children, revealing a negative correlation, which indicated that PA level is related to the clinical typing of children with 2019-nCoV infection. The more obvious the decrease in PA level in the early stage of 2019-nCoV infection is, the severer the condition may be and the longer the hospital stay may be.

At present, there are significant differences in the reported laboratory indicators of children with 2019-nCoV infection. It has been reported that 33% children with 2019-nCoV infection will have LY reduction, 40% will have CRP increase,^21^ and 78% will have PA decrease.^22^ However, it has also been reported that only 3% children present reduced LY and 13.6% have elevated CRP.^23^ Our results showed that only 4.3% of the children presented reduced LY count, 9.3% had increased CRP, and 25% had decreased PA.

### Limitations of this study:

The sample size is small, the proportion of children with asymptomatic infection is high, and the conducted laboratory test items are limited.

## CONCLUSIONS

After the outbreak of this epidemic in Shijiazhuang, the local residents were strictly isolated in time, and the local government actively and repeatedly provided free 2019-nCoV nucleic acid testing, so that all infected children timely received nucleic acid testing, and 2019-nCoV nucleic acid-positive children were actively and effectively treated free. Therefore, most of the included children were mild and asymptomatic.

### Authors’ Contributions:

**JB & YZ:** Carried out the studies, participated in collecting data, drafted the manuscript, and are responsible and accountable for the accuracy and integrity of the work.

**JZ & QH:** Performed the statistical analysis and participated in its design.

**CJ & WZ:** Participated in acquisition, analysis, or interpretation of data and drafting the manuscript.

All authors read and approved the final manuscript.
